# Physiological and image-based characterization of *Botrytis cinerea* infection progression in postharvest ‘Haegeum’ kiwifruit using interpretable deep learning

**DOI:** 10.3389/fpls.2026.1894572

**Published:** 2026-07-27

**Authors:** Joonggon Kim, Sang-Yeon Kim, Kyeonglim Min, Chanyong Jeong, Ghiseok Kim, Eun Jin Lee

**Affiliations:** 1 Department of Agriculture, Forestry and Bioresources, College of Agriculture and Life Sciences, Seoul National University, Seoul, Republic of Korea; 2 Department of Biosystems Engineering, Seoul National University, Seoul, Republic of Korea; 3 Integrated Major in Global Smart Farm, Seoul National University, Seoul, Republic of Korea; 4 Research Institute of Agriculture and Life Sciences, Seoul National University, Seoul, Republic of Korea; 5 Department of Electrical and Computer Engineering, Seoul National University, Seoul, Republic of Korea

**Keywords:** *Actinidia chinensis*, convolutional neural network, disease, firmness, postharvest quality, RGB image

## Abstract

Gray mold caused by *Botrytis cinerea* severely limits the postharvest storage and marketability of kiwifruit, creating a need for accurate and non-destructive infection assessment. In this study, a symptom based, ROI-aware, and interpretable deep-learning framework was developed to classify infection levels in golden-fleshed ‘Haegeum’ kiwifruit. Infection severity was categorized into four levels based on external visual symptoms: Level 1: no lesions; Level 2: slight discoloration; Level 3: localized mycelial growth; Level 4: extensive mycelial growth and tissue breakdown. The associated physiological responses were subsequently characterized for each level. Disease progression induced fruit softening with firmness decreasing from 4.82 at Level 1 to 2.79 N at Level 4, while respiration rates increased from 0.05 to 0.24 µmol kg^-1^ s^-1^. Ethylene production began at Level 3 and sharply increased at Level 4, reaching 0.015 µmol kg^-1^ s^-1^. To quantify lesion severity, a region-of-interest-aware pipeline using RGB blue and LAB L* and a* channels was developed, enabling lesion-focused visualization of infection. Compared with the baseline model, the proposed approach improved classification performance, achieving accuracies of 90.3% with EfficientNet-B0, ResNet-50, and ShuffleNet-V2, and 87.6% with ResNet-18. Gradient-weighted class activation mapping further indicated that the ROI-aware pipeline reduced background-related activation and improved the spatial confinement of model attention within fruit regions. These findings demonstrate that symptom-based RGB classification, supported by *post hoc* physiological characterization and explainable deep-learning analysis, provides a biologically interpretable framework for monitoring visible fungal infection progression in postharvest kiwifruit.

## 1 Introduction

The golden-fleshed ‘Haegeum’ kiwifruit (*Actinidia chinensis* var. *chinensis* cv. ‘Haegeum’) is a commercially important cultivar in Korea, characterized by its yellow flesh and distinct postharvest ripening behavior (Choi et al., 2021; Kim et al., 2023). However, similar to numerous climacteric fruits, it is highly susceptible to fungal infection and rapid quality deterioration during postharvest handling and storage. *Botrytis cinerea* (*B. cinerea*), the causal agent of gray mold, is of particular concern because it can induce latent infections at harvest and rapidly proliferate during storage and distribution, leading to tissue softening and off-flavor development, resulting in substantial economic losses (Dean et al., 2012; Williamson et al., 2007).

Physiologically, ‘Haegeum’ kiwifruit exhibits a typical climacteric ripening pattern characterized by a burst of ethylene production and concomitant increase in respiration rate (Kim et al., 2023). These physiological changes are closely associated with fruit softening and can predispose the fruit to pathogen invasion or accelerate the progression of latent *B. cinerea* infections (Hyun et al., 2022). Consequently, fruit firmness, ethylene production, and CO_2_ evolution are widely recognized as key indicators of both ripening status and disease development (Kim et al., 2017; Niklis et al., 1997; Williamson et al., 2007). Importantly, disease progression in postharvest fruit is a dynamic process in which physiological deterioration and pathogen proliferation are closely intertwined.

Conventional methods for detecting *B. cinerea* rely primarily on visual inspection and destructive laboratory assays, which are labor-intensive and often fail to identify early or asymptomatic infections (Elmer and Michailides, 2007). However, in practical supply chains, the severity of infection, rather than the mere presence or absence of disease, is a critical determinant of marketability and storage life. Despite this importance, objective and non-destructive methods for quantifying infection severity remain limited.

Recent advances in computer vision and deep learning have demonstrated the strong potential of these technologies for the non-destructive assessment of fruit quality in horticultural crops (Wang et al., 2022; Yang and Xu, 2021). Convolutional neural networks (CNNs) have been successfully applied to detect subsurface bruises (Castillo-Girones et al., 2024) and automate multi-trait grading in fruit (Masuda et al., 2023). Furthermore, the integration of explainable artificial intelligence techniques, such as gradient-weighted class activation mapping (Grad-CAM), enables the visualization of model attention, thereby improving the interpretability of classification results (Selvaraju et al., 2017).

However, most existing image-based approaches operate as black-box classifiers and often fail to distinguish biologically relevant features from background artifacts. In particular, the lack of region-of-interest (ROI) constraints and quantitative lesion representation may lead to misinterpretation of disease symptoms, particularly on heterogeneous fruit surfaces. Moreover, such image-based analyses have rarely been linked to the physiological changes that accompany disease progression.

Existing studies on kiwifruit have largely focused on hyperspectral imaging or the detection of general quality attributes (Castillo-Girones et al., 2024; Haghbin et al., 2023), whereas studies that combine quantitative postharvest physiology with Red-Green-Blue (RGB)-based deep learning to classify infection severity levels remain scarce. Because visible symptom development reflects underlying physiological and biochemical changes, jointly examining image-based classification and the associated physiological responses offers a more biologically grounded basis for interpreting disease progression.

Therefore, this study aimed to develop a symptom-based, ROI-aware, and interpretable deep-learning framework for the detection and classification of visible *B. cinerea* infection progression in golden-fleshed ‘Haegeum’ kiwifruit. Infection levels were assigned based on external visual symptoms, whereas postharvest physiological parameters, including fruit firmness, ethylene production, respiration rate, and image-derived infection ratio, were used for *post hoc* characterization of the biological and lesion-related changes associated with each visually assigned level. The proposed approach was evaluated in terms of classification performance and Grad-CAM-based interpretability, providing a non-destructive framework for monitoring visible gray mold infection progression in postharvest kiwifruit.

## 2 Materials and methods

### 2.1 Kiwifruit and experimental design

“Haegeum,” a golden-fleshed kiwifruit (*Actinidia chinensis* var. *chinensis*), was harvested 160 d after full bloom from a commercial orchard in Bosung, Jeollanam-do, South Korea. Fruits were selected based on uniform size and the absence of visible defects, hand-harvested in the morning, and immediately transported to the laboratory. To minimize variability associated with fruit maturity and standardize the physiological baseline among samples, all fruits were preconditioned in cold storage at 0 °C and 85–90% relative humidity for 2.5 months prior to inoculation. The 2.5-month storage period was selected to simulate prolonged commercial cold storage before distribution and marketing, thereby allowing infection progression to be evaluated under commercially relevant postharvest conditions.

The fruits were inoculated with *B. cinerea* spore suspensions. The *B. cinerea* strain BHKA 1-1-1, isolated from ‘Haegeum’ kiwifruit, was cultured on potato dextrose agar (Difco, Detroit, MI, USA) at 25 °C for four weeks to obtain mature sporulating cultures (Hyun et al., 2022). Spore suspensions were prepared in sterile distilled water and adjusted to 3 × 10^8^ spores L^-1^ using a hemocytometer (INCYTO, Cheonan, Korea). A 10-µL droplet of the spore suspension, corresponding to approximately 3 × 10³ spores, was applied to the stylar end of each fruit without wounding.

The inoculated fruits were incubated at 20 °C for 14 d under 80–85% relative humidity in the dark to promote the development of disease. Images were acquired every 2 d during the incubation period to monitor disease progression, and the infection levels were subsequently determined based on visual symptoms.

A total of 240 fruit were used in this experiment and allocated to eight sampling time points (0, 2, 4, 6, 8, 10, 12, and 14 d after inoculation), with 30 fruit evaluated at each time point. At each sampling time point, all 30 fruit were photographed from six views and visually assigned to infection levels based on external symptoms. Subsequently, a subset of 15 photographed fruit was selected for firmness assessment, and 10 photographed fruit were used for respiration rate and ethylene production measurements. The remaining fruit were not used for physiological measurements because they could not be confidently assigned to a specific infection level. This imaging scheme generated 1,440 raw RGB images in total. After image-quality screening, 959 images were retained for model training and evaluation. The experimental design and sample allocation are summarized in **Supplementary Figure S1**.

### 2.2 Fruit firmness measurement

Fruit firmness, expressed in Newtons (N), was determined using a CT-3 texture analyzer (AMETEK Brookfield, Middleboro, MA, USA) fitted with a 4-mm flat probe. After visual infection-level assignment, firmness was measured using fruit showing the corresponding external symptoms during the 14-d incubation period. Each fruit was compressed twice, once on each of two opposite sides of the equatorial region, to a depth of 4 mm per compression at a speed of 1 mm s^-1^ as described by Kim et al. (2023). Firmness was expressed as the mean ± standard error (SE) of 15 biological replicates for each infection level.

### 2.3 Respiration and ethylene measurement

Respiration rate and ethylene production were measured at fixed sampling days after inoculation during the 14-d incubation period. At each sampling day, fruit were visually assigned to the corresponding infection level based on external symptoms before gas measurement. Two kiwifruit were placed in a 1-L airtight plastic jar and held at 25 °C for 2 h. From the headspace, 1 mL of gas was withdrawn and injected into a YL 6400 gas chromatograph system (YOUNGIN Chromass, Anyang, Korea) equipped with a flame ionization detector for ethylene and a thermal conductivity detector for CO_2_ analysis. A Porapak N 80/100 column (Ohio Valley Specialty, Marietta, OH, USA) was used with helium carrier gas at 18 mL min^-1^. Injector and oven temperatures were maintained at 50 °C, and the detector temperatures were set to 200 °C for the thermal conductivity detector and 250 °C for the flame ionization detector. Ethylene and CO_2_ were identified and quantified using external standards. Respiration rate and ethylene production were expressed as the mean ± SE of five biological replicates, corresponding to five sealed jars, each containing two fruit, for each infection level.

### 2.4 Definition of infection levels

**Figure 1:**
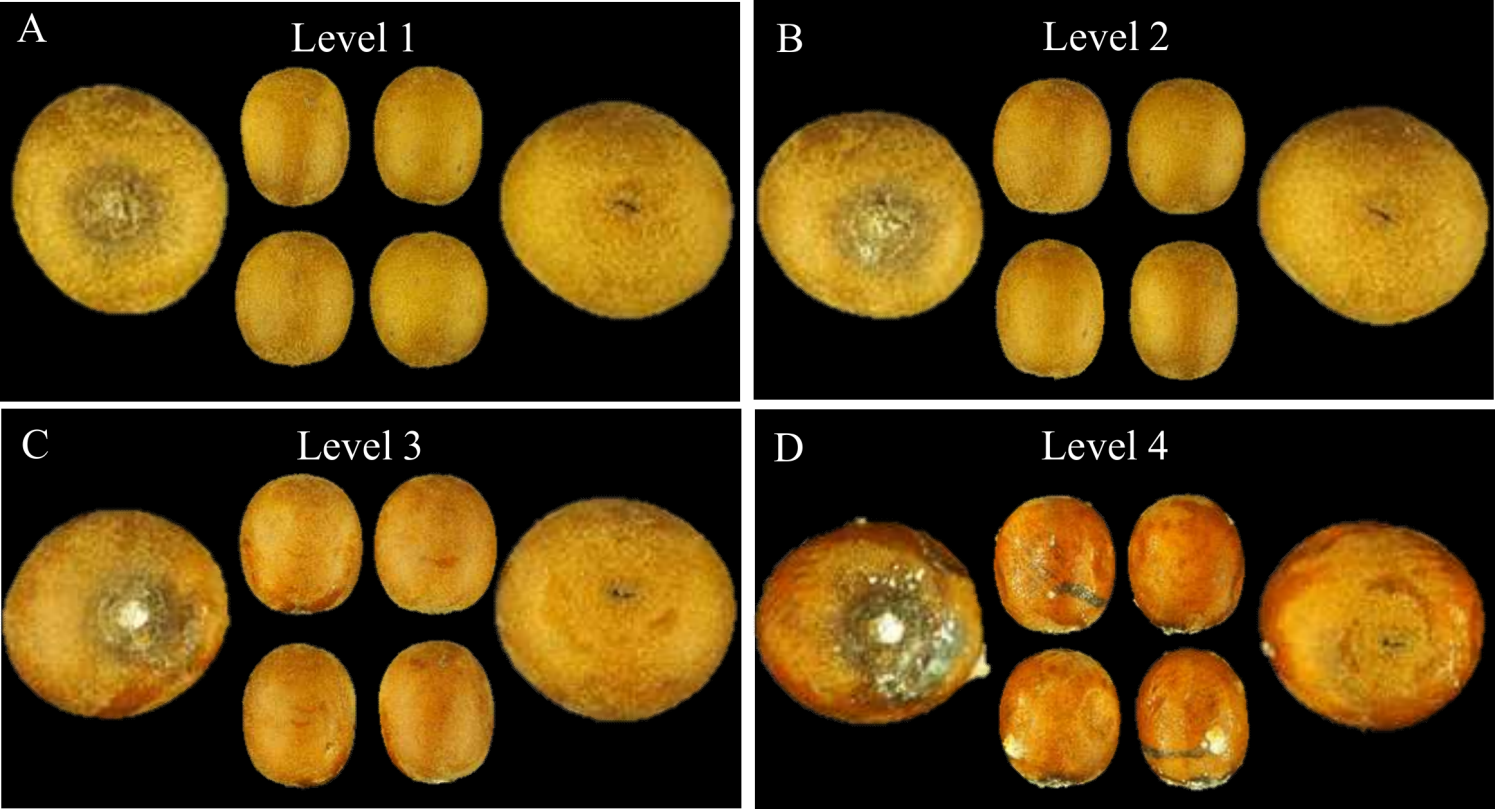
Representative external appearance of ‘Haegeum’ kiwifruit at four visually assigned *Botrytis cinerea* infection levels (Levels 1–4). The levels were assigned based solely on external visual symptoms, not on physiological parameters: **(A)** Level 1, absence of visible lesions; **(B)** Level 2, slight grayish discoloration; **(C)** Level 3, localized white mycelial growth; and **(D)** Level 4, extensive mycelial development and tissue breakdown.

The severity of *B. cinerea* infection was assigned to four levels (Levels 1–4) based solely on external visual symptoms (**Figure 1**). Physiological parameters, including fruit firmness, respiration rate, and ethylene production, were not used as criteria for level assignment. Level 1 was defined as the absence of visible lesions; Level 2 as slight grayish discoloration; Level 3 as localized white mycelial growth; and Level 4 as extensive mycelial development and tissue breakdown. No separate mock-inoculated control group without spores was included; therefore, Level 1 represented inoculated fruit showing no visible lesions at the time of imaging, rather than mock-inoculated healthy fruit. The image-derived infection ratio was also calculated after visual level assignment as a quantitative descriptor of lesion severity, not as a criterion for defining infection levels.

### 2.5 Image acquisition and pre-processing

Six high-resolution RGB images were captured for each fruit (top, bottom, and four evenly spaced lateral views) to obtain a complete 360° representation of the external symptoms. Six-view RGB images of each fruit were acquired under standardized illumination. The same visually assigned infection-level labels described in Section 2.4 were used for both analytical pipelines. In Pipeline I (baseline RGB pipeline), the raw images were resized to 330 × 330 pixels, center-cropped to 224 × 224 pixels, and normalized using the ImageNet mean and standard deviation (SD) prior to model training.

In Pipeline II (ROI-aware pipeline), fruit regions were automatically segmented, and background pixels were masked to zero before model input and Grad-CAM analysis. ROI segmentation was performed using threshold-based foreground separation, followed by morphological refinement, with the largest connected component defined as the fruit region. Subsequent resizing, center-cropping, and normalization were applied identically to the images treated in Pipeline I, ensuring that the only systematic difference between the two pipelines was ROI-based background suppression and infection ratio quantification.

Within each ROI, the infection ratio (%) was quantified using rule-based thresholding of the RGB blue (B) channel in combination with the LAB L* and a* channels. Threshold values were empirically determined from representative images acquired under standardized illumination and then fixed across all samples to ensure reproducibility. Pixels with B > 170 were classified as infected. For pixels with 130 < B < 170, those infected were further identified when L* > 210 or a* < 120, those with 120 < a* < 130 were classified as suspected, and the remaining were classified as healthy. Pixels with B < 130 were classified as healthy. The infection ratio was calculated as the percentage of infected pixels relative to the total ROI pixels using Equation 1.

(1) $$\text{Infection ratio }(\%)=\frac{N_{\text{infected pixels}}}{N_{\text{fruit ROI pixels}}}\times 100$$

This scalar infection ratio was used as a quantitative descriptor of lesion severity for ROI-aware analysis and interpretation of disease progression after visual level assignment, not as a criterion for defining infection levels. The infection ratio was expressed as the mean ± SE for 259, 447, 158, and 95 images corresponding to Levels 1–4, respectively.

### 2.6 Deep learning models and training

Nine deep learning backbones, namely, ResNet-18, ResNet-50, EfficientNet-B0 (Tan and Le, 2019), ShuffleNet-V2 (Ma et al., 2018), MobileNet-V2, MNASNet1_0, GoogLeNet, and Vision Transformer architectures (ViT-Base and ViT-Large), were initially screened. Based on the initial screening of the nine architectures, four CNN backbones (ResNet-18, ResNet-50, EfficientNet-B0, and ShuffleNet-V2) were selected for matched comparison between Pipeline I and Pipeline II because they showed relatively high overall accuracy, macro-averaged precision, recall, and F1-score, together with comparatively balanced class-wise prediction patterns.

For each model, the original classification head was replaced with a fully connected layer that produced four output classes corresponding to the visually assigned infection levels. Only RGB image data were used as model inputs, and physiological parameters, including firmness, respiration rate, and ethylene production, were not used for model training or class prediction. The models were initialized with ImageNet-pretrained weights and fine-tuned on the kiwifruit dataset. Training was performed using stochastic gradient descent (SGD) with an initial learning rate of 0.05, momentum of 0.9, and weight decay of 1 × 10^-4^. These settings followed common SGD-based fine-tuning practices in transfer learning, in which momentum and weight decay are typically fixed to standard values while the learning rate is tuned to the scale and characteristics of the target dataset to balance the preservation of pre-trained representations with sufficient adaptation capacity (Li et al., 2020). A linear learning-rate warm-up was applied over the first five epochs to stabilize the optimization, and all models were trained for 30 epochs.

To ensure fair evaluation and prevent data leakage, all images originating from the same fruit were assigned to the same subset. Model performance was evaluated across three independent fruit-level random splits (train/validation/test = 70:15:15). The checkpoint with the highest validation accuracy in each run was used for the final test evaluation.

The dataset comprised 959 images, distributed as 259, 447, 158, and 95 images for Levels 1–4, respectively. Because the classification task was based on visible symptom patterns and lesion-sensitive color features, data augmentation was limited to random horizontal flipping during training. To preserve the natural lesion distribution characteristics of the dataset, no class-weighted loss or resampling was applied during training. Instead, class imbalance was addressed during the evaluation stage using macro-averaged metrics.

### 2.7 Model evaluation and quantitative metrics

To evaluate both the predictive performance and model interpretability, classical classification metrics and Grad-CAM-based explainability indicators were applied. Accuracy, precision, recall, F1-score, macro-averaged AUC, CAM-based metrics, and FPS were calculated using Equations 2-12).

Accuracy was calculated as the proportion of correctly classified samples using Equation 2:

(2) $$\text{Accuracy}=\;\frac{\sum_{c=1}^{C}TP_{c}}{N}$$

where *TPc* denotes the number of true-positive predictions for class *c*, C denotes the number of infection classes, and N denotes the total number of evaluated images.

Precision, which reflects the reliability of positive predictions, was calculated using Equation 3:

(3) $${\text{Precision}}_{c}=\frac{TP_{c}}{TP_{c}+\;FP_{c}}$$

where *TPc*, *FPc*, and *FNc* denote the true positives, false positives, and false negatives for class *c*, respectively.

Recall, or sensitivity, which represents the proportion of actual positives correctly predicted, was calculated using Equation 4:

(4) $${\text{Recall}}_{c}=\frac{TP_{c}}{TP_{c}+\;FN_{c}}$$

F1-score, the harmonic mean of precision and recall, was calculated using Equation 5:

(5) $$F1_{c}=\frac{2\;\times {\text{ Precision}}_{c}\;\times {\text{ Recall}}_{c}\;}{{\text{Precision}}_{c}+{\text{ Recall}}_{c}}$$

Because the data were not perfectly balanced, macro-averaged metrics were calculated using Equation 6:

(6) $$\begin{array}{c} {\text{Precision}}_{\text{macro}}=\frac{1}{C}\sum_{c=1}^{C}{\text{Precision}}_{c}\\ {\text{Recall}}_{\text{macro}}=\frac{1}{C}\sum_{c=1}^{C}{\text{Recall}}_{c}\\ F1_{\text{macro}}=\frac{1}{C}\sum_{c=1}^{C}F1_{c} \end{array}$$

The macro-averaged area under the curve (AUC), defined as the mean area under the one-vs-rest receiver operating characteristic (ROC) curves, was calculated using Equation 7 to assess threshold-independent class discrimination:

(7) $$AUC_{\text{macro}}=\frac{1}{C}\sum_{c=1}^{C}AUC_{c}$$

where *AUC_c_* is the area under the one-vs-rest *ROC* curve for class *c*.

To describe the spatial distribution of model attention relative to the fruit ROI, Grad-CAM was applied to each test image, and four saliency metrics, including the outside-ROI ratio, entropy within the ROI, CAM area ≥ 0.5, and Drop@20% Δ, were computed. The outside-ROI ratio, calculated using Equation 8 was defined as the proportion of the total CAM activation distributed outside the fruit ROI:

(8) $$\text{Outside ROI ratio}\;(\%)=\frac{\sum_{p\notin \text{ROI}}A(p)}{\sum_{p}A(p)}\times 100$$

Entropy within the ROI, calculated using Equation 9, quantified the spatial dispersion of activation inside the ROI:


$$\tilde{A}(p)=\frac{A(p)}{\sum_{q\in ROI}A(q)}$$

(9) $${\text{Entropy}}_{\text{ROI}}=-\sum_{p\in \text{ROI}}\tilde{A}(p)\log\tilde{A}(p)$$

CAM area at threshold 0.5, calculated using Equation 10, was defined as the fraction of ROI pixels with normalized CAM intensity ≥ 0.5:

(10) $${\text{CAM area}}_{\geq 0.5}=\frac{\left|\{p\in \text{ROI}\;:A(p)\;\geq \;0.5\}\right|}{\left|\text{ROI}\right|}$$

In addition, a patch-based confidence deletion test was performed to examine the sensitivity of model confidence to CAM-highlighted regions. For each test image, the top 20% CAM-highlighted region within the ROI was masked, and the resulting probability drop was compared with that obtained when a random region of equal size within the ROI was masked instead. The probability drop and Drop@20% Δ metric were calculated using Equation 11:

(11) $$\begin{array}{c} P_{\text{drop}}(\cdot )=P_{\text{original}}-P_{\text{masked}}\\ \text{Drop}@20\text{\% Δ}=P_{\text{drop}}(\text{CAM})-P_{\text{drop}}(\text{Random}) \end{array}$$

where $P_{\text{original}}$ and $P_{\text{masked}}$ denote the predicted probabilities of the true class before and after masking, respectively.

Therefore, positive values of Drop@20% Δ suggest that CAM-highlighted regions contributed more to model confidence than randomly selected regions of the same size. However, this deletion-based metric was interpreted as complementary evidence of prediction sensitivity rather than definitive causal validation of model attention. These combined performance and saliency metrics enabled a direct comparison of Pipeline I (baseline RGB pipeline) and Pipeline II (ROI-aware pipeline) in terms of classification performance, attention confinement, and computational efficiency.

To evaluate computational efficiency during inference, model speed was measured using frames per second (FPS), calculated using Equation 12:

(12) $$\text{FPS}=\;\frac{N_{\text{images}}}{T_{\text{inference}}}$$

where *N*_images_​ is the total number of test images processed and *T*_inference_ (s) is the total forward-pass time required to classify those images.

FPS was measured in the inference-only mode using the test data loader under identical hardware conditions.

### 2.8 Statistical analysis

The physiological measurements and image-derived infection ratio were analyzed under a completely randomized design. Physiological data and the infection ratio were expressed as the mean ± SE, whereas model performance metrics derived from three independent fruit-level random splits were expressed as the mean ± SD. SE was used for physiological variables and the infection ratio to describe the precision of the estimated mean response for each visually assigned infection level, whereas SD was used for model-performance metrics to represent variability among repeated fruit-level data splits. Differences in firmness, respiration rate, ethylene production, and infection ratio among visually assigned infection levels (Levels 1–4) were evaluated by one-way analysis of variance, followed by Tukey’s honestly significant difference test for multiple comparisons at p < 0.05. These statistical comparisons were used to characterize physiological and lesion-related differences associated with visually assigned levels, not to define or validate the infection classes. For the matched comparison of the four selected CNN backbones, model performance and CAM-based quantitative metrics were expressed as the mean ± SD across three independent fruit-level repeated splits.

Statistical analyses were performed using IBM SPSS Statistics 21 (IBM, Armonk, NY, USA). A p-value of < 0.05 was considered statistically significant.

## 3 Results

### 3.1 Physiological characterization of visually assigned *B. cinerea* infection levels

After visual assignment of infection levels, physiological measurements were used to characterize the biological changes associated with *B. cinerea* infection progression in ‘Haegeum’ kiwifruit (**Table 1**; **Figure 1**). Fruit firmness consistently decreased from 4.82 N at Level 1 to 2.79 N at Level 4, indicating progressive softening associated with infection development. The respiration rate increased from 0.05 µmol kg^-1^ s^-1^ at Level 1 to 0.21 µmol kg^-1^ s^-1^ at Level 2, followed by a transient decline at Level 3 (0.12 µmol kg^-1^ s^-1^) and a subsequent increase to 0.24 µmol kg^-1^ s^-1^ at Level 4, forming a climacteric-like pattern. Ethylene production was not detected at early stages (Levels 1 and 2) but became detectable at Level 3 and increased sharply to 0.015 µmol kg^-1^ s^-1^ at Level 4 (**Table 1**). These results indicate that visually assigned infection levels were accompanied by ripening-like physiological responses, including enhanced respiration and ethylene biosynthesis, accompanied by substantial tissue softening. After visual level assignment, the image-derived infection ratio was used as a quantitative descriptor of lesion severity and numerically increased from 0.13% at Level 1 to 4.50% at Level 4 (**Table 1**), indicating an increase in lesion burden within the fruit ROI. A descriptive heatmap based on level-wise mean values is provided in **Supplementary Figure S2** only as a supplementary visualization of the trends summarized in **Table 1**.

**Table 1:** Physiological characterization and image-derived infection ratio of visually assigned *Botrytis cinerea* infection levels in ‘Haegeum’ kiwifruit.

Infection level	Firmness (N)	Respiration rate (µmol kg^-1^ s^-1^)	Ethylene production (µmol kg^-1^ s^-1^)	Infection ratio (%)
Level 1	4.82 ± 0.24d	0.05 ± 0.01a	n.d. a	0.13 ± 0.00a
Level 2	3.79 ± 0.12c	0.21 ± 0.01c	n.d. a	0.33 ± 0.00a
Level 3	3.30 ± 0.07b	0.12 ± 0.01b	0.001 ± 0.001b	0.94 ± 0.01a
Level 4	2.79 ± 0.13a	0.24 ± 0.02c	0.015 ± 0.002c	4.50 ± 3.06b

The values are expressed as the mean ± SE of 5 replicates for respiration and ethylene production and 15 replicates for firmness for each visually assigned infection level. For respiration and ethylene production, five biological replicates corresponded to five sealed jars, each containing two fruit, for a total of 10 fruit per infection level. N denotes Newtons, the unit of fruit firmness. The infection ratio was expressed as the mean ± SE of 259, 447, 158, and 95 images corresponding to Levels 1, 2, 3, and 4, respectively. The infection ratio was calculated after visual level assignment and was used as a quantitative descriptor of lesion severity, not as a criterion for defining infection levels. Different letters in the same column for firmness, respiration rate, ethylene production, and infection ratio indicate significant differences among infection levels (p < 0.05) according to one-way analysis of variance, followed by Tukey’s HSD test. n.d., not detected.

### 3.2 Simplified workflow and ROI-aware infection-ratio representation

**Figure 2:**
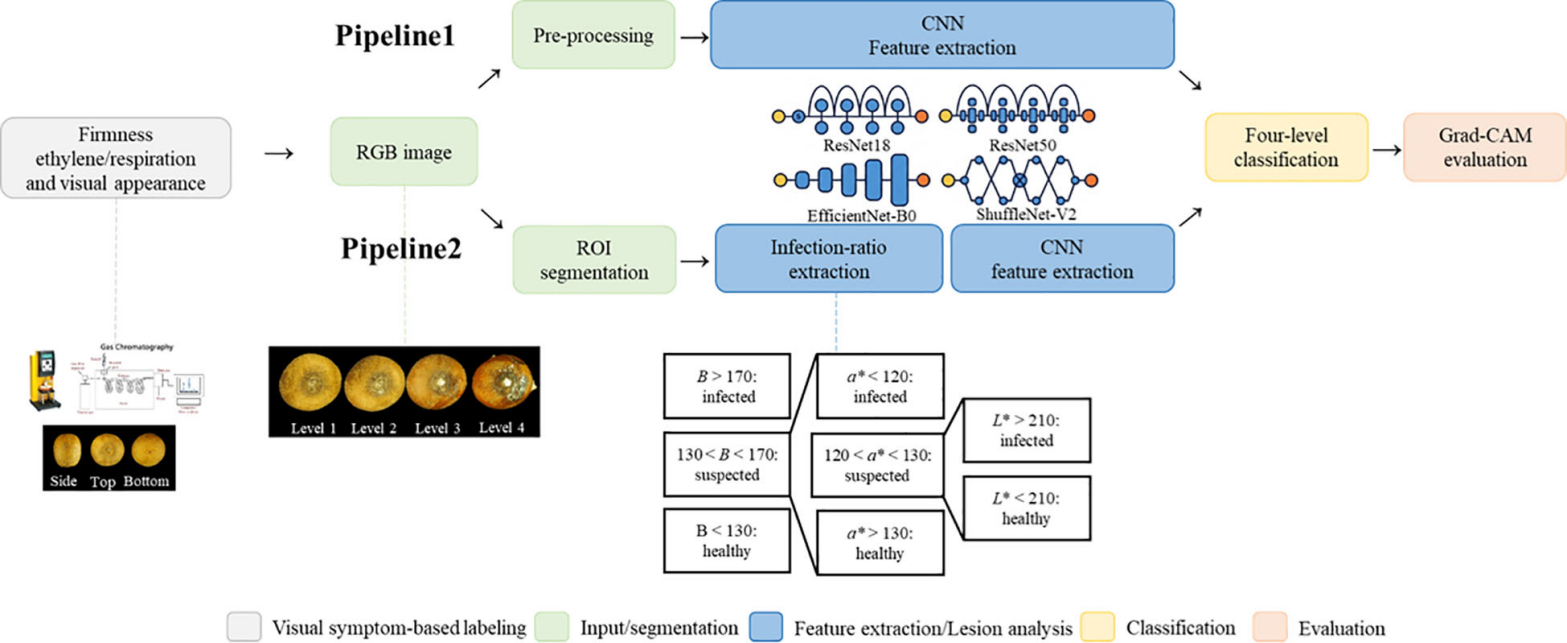
Simplified workflows of Pipeline I and Pipeline II for the four-level classification of visible *Botrytis cinerea* infection progression in ‘Haegeum’ kiwifruit. Infection levels (Levels 1–4) were assigned based solely on external visual symptoms, and physiological parameters were used only for subsequent characterization of infection progression. Pipeline I employed standard RGB preprocessing, followed by CNN-based feature extraction, classification, and Grad-CAM evaluation. Pipeline II additionally incorporated automatic fruit ROI segmentation and infection-ratio extraction using the RGB blue channel in combination with the LAB L* and a* channels prior to CNN-based feature extraction and evaluation. Representative CNN backbones are schematically illustrated.

The simplified workflows and key differences between Pipeline I (baseline RGB pipeline) and Pipeline II (ROI-aware pipeline) are presented in **Figure 2**. Threshold values for infection ratio extraction were empirically determined from representative images acquired under standardized illumination and subsequently fixed for all samples to ensure consistent lesion discrimination. In Pipeline I, images with visually assigned infection-level labels were used without explicit segmentation, whereas in Pipeline II, automatic fruit ROI masking was performed before model input and Grad-CAM analysis, and the infection ratios were calculated using rule-based thresholding of the RGB blue channel in combination with the LAB L* and a* channels. This preprocessing reduced non-informative background features, such as tray edges and specular reflections, and provided a fruit-region-constrained input representation.

The combined use of the LAB L* and a* channels with the RGB blue channel enabled threshold-based discrimination of infected, suspected, and healthy pixels within the fruit ROI. The infection ratio, defined as the proportion of infected pixels within the segmented ROI, was used as a quantitative lesion descriptor after visual level assignment, rather than as a criterion for defining infection levels. Inspection of the ROI masks indicated effective background suppression and improved contrast between healthy and infected tissues, resulting in a constrained input representation for subsequent CNN training and CAM-based analysis.

### 3.3 Classification performance in the preliminary screening stage

The overall classification performance of the nine deep learning backbones is summarized in **Table 2**, and the class-wise prediction patterns for Pipeline I and II are presented in **Supplementary Figures S3**, **S4**. A confusion matrix analysis showed that Pipeline II (ROI-aware pipeline) reduced misclassification between adjacent infection levels in several CNN models, whereas MNASNet1_0 exhibited inconsistent class separation (**Supplementary Figure S5**).

**Table 2:** Comparative classification metrics (accuracy, precision, recall, and F1-score) of *Botrytis cinerea* infection levels in ‘Haegeum’ kiwifruit across two experimental pipelines (Pipeline I vs. Pipeline II).

Model	Pipeline	Accuracy (%)	Precision (%)	Recall (%)	F1-score (%)
EfficientNet-B0	I	69.0	59.8	59.3	56.6
	II	90.3	93.7	91.8	92.1
GoogLeNet	I	71.7	66.9	65.0	63.8
	II	75.2	71.9	70.6	71.3
MNASNet1_0	I	55.9	51.0	52.7	42.1
	II	50.3	50.0	50.0	50.0
MobileNet-V2	I	66.9	57.2	59.7	56.6
	II	75.2	75.0	65.6	69.5
ResNet18	I	73.8	65.0	72.0	70.8
	II	87.6	87.5	80.8	83.8
ResNet50	I	75.2	82.2	77.8	78.2
	II	90.3	90.6	90.6	90.6
ShuffleNet-V2	I	69.0	62.3	52.3	57.4
	II	90.3	92.1	92.3	92.1
ViT-Base	I	72.4	65.6	64.5	62.1
	II	77.9	78.1	77.3	77.7
ViT-Large	I	71.7	74.4	74.1	74.4
	II	71.7	71.9	73.1	73.1

Among the nine evaluated architectures (ResNet-18, ResNet-50, EfficientNet-B0, ShuffleNet-V2, MNASNet1_0, MobileNet-V2, GoogLeNet, ViT-Base, and ViT-Large), the classification performance was generally higher in Pipeline II than in Pipeline I, although improvements varied among models. In Pipeline II, EfficientNet-B0, ResNet-50, and ShuffleNet-V2 each achieved an accuracy of 90.3%, whereas ResNet-18 achieved 87.6%. In contrast, MNASNet1_0 showed lower performance (50.3%) (**Table 2**), indicating that the effectiveness of the ROI-aware pipeline depends on the model architecture and feature extraction capability.

The precision–recall balance also became more stable across most CNN models. ResNet-18, ResNet-50, EfficientNet-B0, and ShuffleNet-V2 demonstrated the highest and most consistent performance and were selected for further analysis. Across both analytical pipelines, these CNN-based models consistently achieved higher classification performance than the vision transformer models evaluated in this study (**Table 2**; **Supplementary Figure S5**). Among these, EfficientNet-B0, ResNet-50, and ShuffleNet-V2 showed comparable classification performance, each reaching 90.3% accuracy, indicating that the model architecture had a limited influence on the final accuracy under Pipeline II. ShuffleNet-V2 achieved a similar performance while requiring fewer computations, underscoring that lightweight models can achieve high precision when coupled with ROI-aware preprocessing and lesion-sensitive image representation. ResNet-50, despite its higher parameter count, did not outperform other CNN models, and ResNet-18 showed slightly lower performance (87.6%), suggesting that increasing network depth does not necessarily improve classification accuracy when visible lesion-related features are spatially constrained within the fruit ROI (**Table 2**).

Collectively, these preliminary screening results indicate that ROI-based preprocessing and lesion-ratio representation, rather than the network complexity alone, contributed to improved predictive accuracy. These result motivated the selection of four CNN backbones for the matched comparison presented in the following sections.

### 3.4 ROC analysis and class separability in the matched comparison

**Figure 3:**
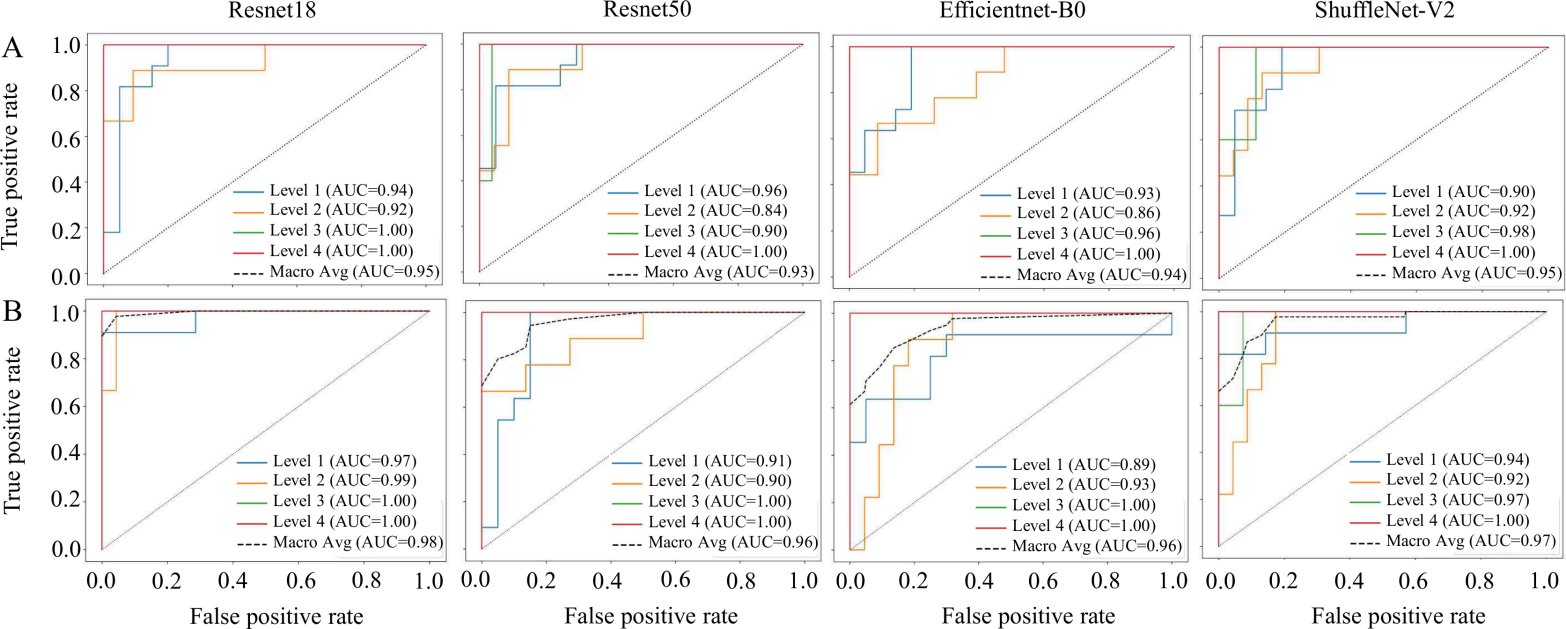
ROC curves for multi-class classification of visually assigned *Botrytis cinerea* infection levels (Levels 1–4) in ‘Haegeum’ kiwifruit across four CNN backbones. **(A)** Pipeline I (baseline pipeline without ROI masking or infection-ratio control); **(B)** Pipeline II (ROI-aware pipeline with background suppression and infection-ratio quantification based on RGB and LAB channels). Each panel corresponds to a backbone (ResNet-18, ResNet-50, EfficientNet-B0, and ShuffleNet-V2). The solid lines represent class-wise ROC curves for Levels 1–4, and the dashed line indicates the macro-average.

A ROC analysis was performed to compare threshold-independent class separability between Pipeline I (baseline RGB pipeline) and Pipeline II (ROI-aware pipeline) (**Figure 3**). In the matched comparison, AUCmacro increased in all four selected CNN backbones under Pipeline II, rising from 94.89 ± 0.02 to 98.13 ± 0.02 in ResNet-18, from 93.08 ± 0.09 to 96.02 ± 0.02 in ResNet-50, from 93.82 ± 0.07 to 95.50 ± 0.01 in EfficientNet-B0, and from 94.57 ± 0.02 to 96.55 ± 0.01 in ShuffleNet-V2. These results indicate that the ROI-aware pipeline improved class separability, with the clearest gain observed in ResNet-18, while maintaining high discrimination in the other three backbones.

### 3.5 Grad-CAM interpretability and computational efficiency in ‘Haegeum’ kiwifruit

**Figure 4:**
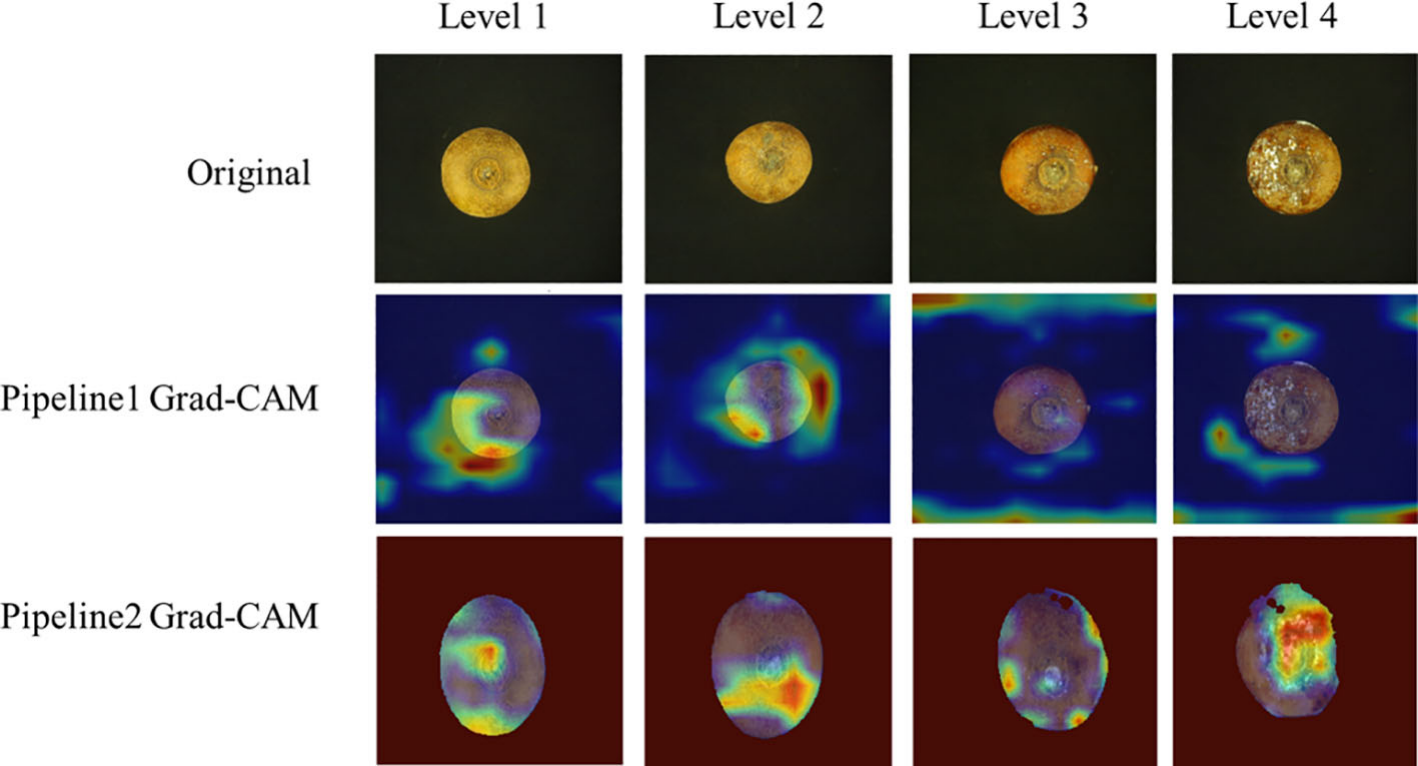
Representative Grad-CAM activation maps for identical ‘Haegeum’ kiwifruit samples across four visually assigned *Botrytis cinerea* infection levels (Levels 1–4) using ResNet-18. Each column corresponds to an infection level. Top row: original RGB images. Middle row: Grad-CAM overlays from Pipeline I (baseline pipeline without ROI masking), showing substantial activation extending outside the fruit region. Bottom row: Grad-CAM maps from Pipeline II (ROI-aware pipeline), showing more spatially confined activation within the fruit region. The progressive shift in saliency distribution from background-dominated patterns in Pipeline I to fruit-focused patterns in Pipeline II illustrates the effect of ROI-aware preprocessing on model attention patterns, rather than definitive causal localization of infection-related regions.

**Figure 5:**
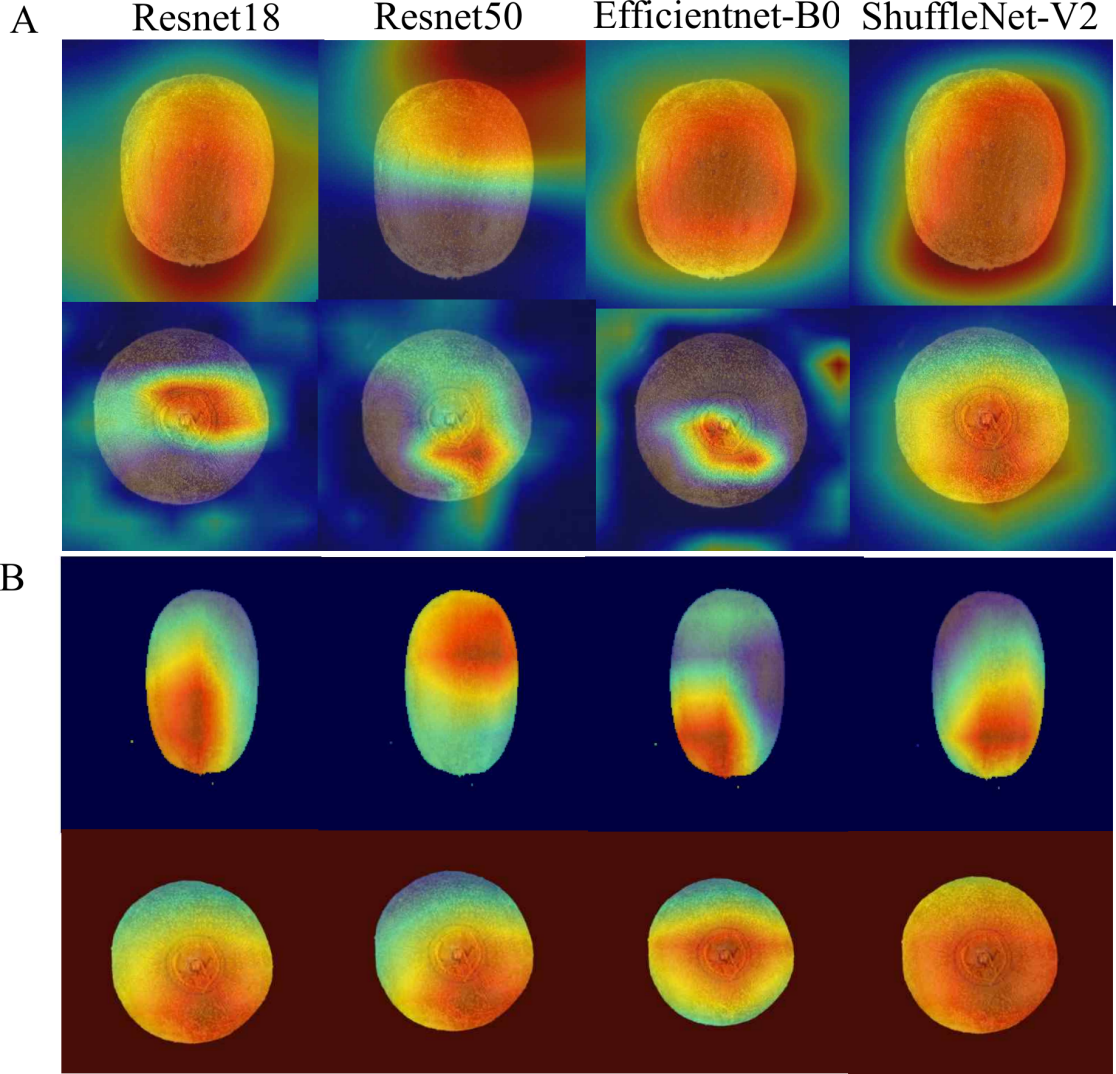
Representative Grad-CAM activation maps across four CNN backbones (ResNet-18, ResNet-50, EfficientNet-B0, and ShuffleNet-V2) for visually assigned *Botrytis cinerea* infection classification in ‘Haegeum’ kiwifruit. **(A)** Pipeline I (baseline pipeline without ROI masking or infection-ratio control). **(B)** Pipeline II (ROI-aware pipeline with background suppression and infection-ratio quantification based on RGB and LAB channels). Each panel shows a representative infected sample. Images within each Pipeline were derived from the same sample to enable a direct cross-backbone comparison of the saliency distributions.

Grad-CAM visualization and quantitative evaluation provided additional insights into model interpretability in the matched comparison (**Figures 4**, **5**; **Table 3**). Pipeline II reduced the outside-ROI ratio across all four selected CNN backbones, indicating that ROI-aware preprocessing reduced background-related activation and improved the spatial confinement of saliency within the fruit region. The outside-ROI ratio decreased from 0.77 ± 0.08 to 0.59 ± 0.16 in ResNet-18, from 0.78 ± 0.05 to 0.48 ± 0.13 in ResNet-50, from 0.71 ± 0.19 to 0.32 ± 0.07 in EfficientNet-B0, and from 0.51 ± 0.03 to 0.26 ± 0.06 in ShuffleNet-V2 (**Table 3**). Entropy within the ROI also decreased in all four backbones, indicating more spatially concentrated saliency distributions in Pipeline II. CAM area ≥ 0.5 decreased in all four backbones, showing that high-intensity activation became more spatially compact rather than more broadly distributed within the ROI.

**Table 3:** Matched comparison of the macro-averaged AUC (*AUC*_macro_), computational efficiency (FPS), and CAM-based quantitative metrics (outside-ROI ratio, entropy within ROI, CAM area _≥ 0.5_, and Drop@20% Δ) of four CNN backbones (ResNet-18, ResNet-50, EfficientNet-B0, and ShuffleNet-V2) for the prediction of the *Botrytis cinerea* infection level in ‘Haegeum’ kiwifruit across Pipeline I (baseline RGB pipeline) and Pipeline II (ROI-aware pipeline).

Model	Pipeline	*AUC*_macro_	FPS	Outside-ROI ratio	Entropy within ROI	CAM area _≥ 0.5_ (%)	Drop@20% Δ
ResNet18	I	94.89 ± 0.02	16.66 ± 1.23	0.77 ± 0.08	13.39 ± 0.50	0.38 ± 0.49	−0.08 ± 0.03
	II	98.13 ± 0.02	17.65 ± 0.68	0.59 ± 0.16	12.15 ± 0.78	0.17 ± 0.10	−0.17 ± 0.11
ResNet50	I	93.08 ± 0.09	11.44 ± 0.27	0.78 ± 0.05	13.66 ± 0.20	0.45 ± 0.38	+0.10 ± 0.07
	II	96.02 ± 0.02	12.31 ± 0.06	0.48 ± 0.13	12.46 ± 0.77	0.25 ± 0.12	+0.04 ± 0.03
EfficientNet-B0	I	93.82 ± 0.07	17.26 ± 1.06	0.71 ± 0.19	13.42 ± 0.41	0.52 ± 0.15	+0.06 ± 0.02
	II	95.50 ± 0.01	17.06 ± 0.26	0.32 ± 0.07	12.98 ± 0.21	0.45 ± 0.19	−0.05 ± 0.01
ShuffleNet-V2	I	94.57 ± 0.02	21.48 ± 0.92	0.51 ± 0.03	13.84 ± 0.04	0.77 ± 0.04	−0.04 ± 0.01
	II	96.55 ± 0.01	21.43 ± 0.30	0.26 ± 0.06	12.61 ± 0.25	0.19 ± 0.10	+0.06 ± 0.03

The values are presented as the mean ± SD (n = 3 fruit level repeated splits). CAM area _≥ 0.5_ (%) indicates the proportion of ROI pixels with normalized CAM intensity ≥ 0.5, and Drop@20% Δ denotes the confidence drop after CAM-guided masking minus that after random masking at 20% ROI deletion (CAM−Random).

The Drop@20% confidence-deletion analysis did not show uniform improvement. ShuffleNet-V2 changed from −0.04 ± 0.01 in Pipeline I to +0.06 ± 0.03 in Pipeline II, whereas ResNet-50 remained positive in both pipelines but decreased from +0.10 ± 0.07 to +0.04 ± 0.03. In contrast, ResNet-18 and EfficientNet-B0 showed more negative Drop@20% Δ values in Pipeline II. These results indicate that Pipeline II consistently improved spatial saliency confinement; however, its effect on confidence-deletion sensitivity remained selective and backbone-dependent (**Table 3**). Given that these metrics were obtained from only three repeated splits, the Grad-CAM and deletion-based metrics should be interpreted as directional, complementary indicators of model attention and prediction sensitivity rather than definitive causal validation of infection-related regions.

The computational efficiency, evaluated as FPS, showed only modest differences between the two pipelines. ShuffleNet-V2 remained the fastest backbone in both pipelines, recording 21.48 ± 0.92 FPS in Pipeline I and 21.43 ± 0.30 FPS in Pipeline II, whereas ResNet-50 remained the slowest at 11.44 ± 0.27 and 12.31 ± 0.06 FPS, respectively. ResNet-18 showed a slight increase from 16.66 ± 1.23 to 17.65 ± 0.68 FPS, whereas EfficientNet-B0 remained nearly unchanged (17.26 ± 1.06 vs. 17.06 ± 0.26 FPS). Thus, under the matched comparison, the principal advantage of Pipeline II lay in reducing activation related to background and improving saliency confinement within the fruit ROI rather than in dramatically increasing inference speed (**Figures 4**, **5**; **Table 3**).

The representative Grad-CAM maps shown in **Figure 4** further indicated that the shift from background-including activation to fruit-focused activation occurred consistently across infection levels in ResNet-18, whereas **Figure 5** showed that this ROI-constraining effect also occurred across the four selected backbone architectures. Together, these findings demonstrate that the refined pipeline improved spatial saliency confinement within the fruit ROI, whereas improvements in deletion-based interpretability and computational efficiency were selective rather than uniform across backbones.

## 4 Discussion

This study developed a symptom-based, ROI-aware, and interpretable deep-learning framework for classifying visible *B. cinerea* infection progression in golden-fleshed ‘Haegeum’ kiwifruit using ROI-aware RGB image analysis supported by *post hoc* physiological characterization (**Table 1**; **Figure 2**). Infection levels were assigned based on external visual symptoms, whereas fruit firmness, respiration rate, ethylene production, and the image-derived infection ratio were used for *post hoc* characterization of physiological and lesion-related changes. These parameters were not used as criteria for class definition or as model inputs. Unlike conventional image-based classification approaches, the proposed framework used ROI-based preprocessing and an image-derived infection-ratio descriptor to provide an interpretable image-based description of visible disease progression.

Progressive fungal infection was accompanied by physiological deterioration, including firmness loss, elevated respiration rate, and late-stage ethylene production (**Table 1**). These responses are indicative of climacteric-like metabolic activation and are consistent with previous reports on pears and plums, where fungal infection and tissue damage stimulated ethylene biosynthesis and cell-wall degradation during senescence-related ripening processes (Castillo-Girones et al., 2024; Li et al., 2025). Similar responses have been reported in kiwifruit, in which *B. cinerea* infection accelerated ethylene production and promoted premature ripening during storage (Niklis et al., 1997). Collectively, these findings indicate that fungal invasion can be associated with host metabolic activity and tissue disintegration, ultimately resulting in visible discoloration and structural collapse of infected tissues (**Figure 1**). Overall, the physiological measurements summarized in **Table 1** indicate that visually assigned infection levels were accompanied by fruit softening, increased respiration, and ethylene production at advanced infection stages. These responses were used to characterize biological changes associated with visible symptom progression, not to define or validate the infection classes.

At the feature-extraction level, the combined use of RGB blue and LAB L* and a* channels provided a rule-based image representation for separating visually infected, suspected, and healthy regions within the fruit ROI under standardized imaging conditions. In particular, the L* and a* channels were useful for representing tissue darkening, discoloration, and surface desaturation associated with lesion progression, consistent with the perceptual uniformity and color-separation advantages of the CIE 1976 color space (McLaren, 1976). Similar benefits of LAB-space integration have been reported in fruit maturity and quality assessment studies, where fused color-space representations improved the discrimination of subtle surface variations under heterogeneous imaging conditions (Masuda et al., 2023; de Moraes et al., 2024). In this study, the RGB/LAB-based infection-ratio representation was used as an image-derived lesion descriptor after visual level assignment, rather than as an independently validated ground-truth annotation of lesion area. Therefore, further validation against manual lesion annotations and additional imaging conditions will be required to improve the robustness and generalizability of this threshold-based approach.

A comparison of Pipeline I and Pipeline II further demonstrated that the preprocessing strategy substantially influenced both classification performance and model attention patterns (**Figure 2**). In Pipeline II, ROI-aware preprocessing reduced non-informative background components, including tray reflections, shadows, and illumination gradients, thereby reducing the saliency leakage outside the fruit region. This result highlights the importance of segmentation-based preprocessing in horticultural imaging and is consistent with previous findings that ROI-constrained preprocessing can improve the extraction of crop-related visual features from complex visual environments (Ni et al., 2020).

A direct comparison between Pipeline I and Pipeline II further suggested that the ROI-aware framework was particularly advantageous for preliminary model screening and spatial refinement of CNN attention (**Table 2**). Pipeline II generally improved the classification performance across architectures and tended to favor CNN-based backbones over transformer-based models. Notably, lightweight architectures such as EfficientNet-B0 and ShuffleNet-V2 achieved performance comparable to that of ResNet-50, despite substantially smaller parameter sizes, supporting previous reports that lightweight CNNs can achieve competitive accuracy with superior computational efficiency (Sandler et al., 2018; Shahi et al., 2022).

Nevertheless, the effects of ROI-aware preprocessing were selective rather than universally beneficial. The most consistent outcome observed in Pipeline II was the reduction in saliency activation outside the fruit ROI, whereas improvements in deletion-based interpretability and classification accuracy varied depending on the backbone architecture (**Figures 4**, **5**; **Table 3**). Similarly, although the *AUC*_macro_ values generally increased across the CNN backbones, the magnitude of improvement differed among the models (**Figure 3**; **Table 3**). These findings suggest that ROI-aware preprocessing primarily improved attention confinement within the fruit ROI, rather than guaranteeing a uniformly superior predictive performance. Therefore, Pipeline II should be interpreted principally as an ROI-constraining and saliency-refinement framework.

Class-wise prediction patterns further revealed that severe infection stages (Levels 3–4) were more readily recognized by the better-performing CNN backbones (**Supplementary Figure S5**). In contrast, the early stages, particularly Levels 1 and 2, were more difficult to separate because Level 1 represented inoculated fruit without visible lesions at the time of imaging, whereas Level 2 showed only slight grayish discoloration. Such subtle visual differences may overlap with natural variation in fruit surface color, illumination, and early symptom development, leading to potential confusion between asymptomatic or weakly symptomatic fruit and early visible infection. Advanced infection stages exhibited clearly visual symptoms, including mycelial growth, tissue collapse, and extensive discoloration, which likely facilitated robust feature extraction from RGB images (**Figure 1**). However, because these advanced stages can often be identified by visual inspection, the practical value of classifying Levels 3 and 4 should be interpreted with caution. In the present study, the four-level classification framework was therefore intended primarily to establish a structured severity-grading dataset and to evaluate whether RGB-based models could separate progressive visible symptom stages under controlled imaging conditions, rather than to replace human recognition of advanced decay.

Because the classification task relied on localized lesion structures, subtle discoloration, and fine-scale texture degradation within the fruit ROI, CNN-based architectures possessing a strong local inductive bias were better suited to capture these spatially localized patterns than transformer-based models. This characteristic likely contributed to the superior CNN performance observed in this study (**Table 2**, **Supplementary Figure S5**). Although vision transformers have demonstrated strong performance in large-scale image-recognition tasks, their effectiveness may be limited in relatively small datasets with insufficient local inductive bias, where CNNs are often advantageous in modeling localized spatial structures (Akkaya et al., 2024). Furthermore, the relatively limited dataset size and minimal augmentation strategy employed in this study may have further constrained transformer performance. In this study, data augmentation was applied conservatively because the classification task depended on subtle symptom-related features, including slight grayish discoloration, localized mycelial development, lesion texture, and RGB/LAB-derived color information. Strong geometric or color-based augmentation could have artificially altered these biologically relevant symptom characteristics and weakened the interpretability of the image-derived lesion descriptors. However, this conservative augmentation strategy may have limited the diversity of training samples and reduced model robustness under variable imaging conditions. Future studies should therefore evaluate symptom-preserving augmentation strategies that improve model generalization without distorting visible infection characteristics.

In addition, Grad-CAM analyses suggested that ROI-aware preprocessing improved the spatial consistency of model attention rather than uniformly enhancing interpretability. Saliency maps became more spatially confined to fruit ROI and visible lesion-related regions, whereas deletion-based causal relevance remained architecture dependent. This observation indicates that spatial concentration and causal importance represent related but distinct dimensions of interpretability (Adebayo et al., 2018; Petsiuk et al., 2018). Therefore, although ROI-aware preprocessing can guide CNN attention toward visible lesion-related regions within the fruit ROI, the spatial alignment of saliency alone should not be interpreted as definitive evidence of a reliable causal explanation. Consequently, consistent with previous interpretability studies, the Grad-CAM-based metrics used in this study should be considered complementary descriptors of model attention rather than direct measures of explanatory validity (Adebayo et al., 2018; Hooker et al., 2019).

Nevertheless, the spatial correspondence between Grad-CAM activation and visible symptom-related regions was consistent with the physiological trends observed after visual level assignment (**Table 1**; **Figures 4**, **5**). Strong activations generally coincided with visually softened, discolored, or mycelium-affected tissues, suggesting that the CNNs learned lesion-associated texture and color features related to visible disease progression. However, this relationship should be interpreted as indicating consistency with physiological trends rather than serving as a direct one-to-one validation against individual physiological measurements.

Importantly, ROI-aware preprocessing did not substantially reduce the inference throughput across the evaluated CNN backbones (**Table 3**), indicating that improved attention confinement within the fruit ROI was achieved without meaningful computational penalties. From a practical perspective, this finding suggests that the primary advantage of the proposed framework lies in its ability to reduce background-related activation and improve spatial attention confinement while maintaining deployment efficiency. Accordingly, backbone selection for practical applications should consider the balance among predictive performance, interpretability, and throughput, rather than classification accuracy alone (Rojas Santelices et al., 2025).

Among the evaluated architectures, ShuffleNet-V2 demonstrated a favorable deployment-oriented profile by combining high classification performance, reduced outside-ROI activation, and the highest inference speed (**Figure 5**; **Table 3**). Although ShuffleNet-V2 also showed a positive Drop@20% Δ value under Pipeline II, this result should be interpreted as complementary evidence of prediction sensitivity rather than definitive causal validation of model attention. This finding is consistent with recent fruit-imaging studies emphasizing that lightweight architectures can provide a practical balance between accuracy and computational cost in agricultural AI systems (Mondal et al., 2025; Rojas Santelices et al., 2025).

Finally, the combined use of RGB and LAB color information provides a lower-cost and simpler image-based approach compared with high-cost hyperspectral imaging systems. Recent work by Gutiérrez et al. (2026) further supports the relevance of ROI-based image analysis for plant disease detection, showing that classifiers trained on ROI-derived hyperspectral and multicolor fluorescence data could detect *B. cinerea*-associated soft rot in melon leaves. While that study focused on early disease detection in leaves using advanced spectral sensors, the present study applied RGB/LAB-based features to visible infection grading in postharvest kiwifruit. Together, these findings emphasize the value of region-focused image information for disease classification across different crops, tissues, and imaging modalities. Although, hyperspectral imaging offers excellent sensitivity for early disease detection (Haghbin et al., 2023), its complexity and cost limit its scalability in commercial postharvest environments. In contrast, the proposed ROI-aware RGB/LAB framework generated an image-derived lesion representation under standardized imaging conditions using standard digital imaging and simple color-based features. However, the present RGB/LAB threshold-based infection-ratio approach should be further validated against manual lesion annotations, external harvest seasons, orchard conditions, imaging environments, and naturally infected fruit before practical deployment. Another limitation of the present dataset is that it did not include a separate mock-inoculated healthy control group. Therefore, Level 1 should be interpreted as inoculated fruit without visible lesions at the time of imaging, rather than as non-inoculated healthy fruit. Future studies should include mock-inoculated healthy fruit, naturally healthy fruit, and naturally infected fruit collected under commercial storage and distribution conditions, with particular emphasis on improving the detection of asymptomatic or early-stage infection and the discrimination between healthy fruit and Level 2 or pre-symptomatic infection.

## 5 Conclusion

This study developed a symptom-based, ROI-aware, and interpretable deep-learning framework for the non-destructive classification of visible *B. cinerea* infection progression in golden-fleshed ‘Haegeum’ kiwifruit. Infection levels were assigned based on external visual symptoms, while physiological parameters and the image-derived infection ratio were used for *post hoc* characterization of biological and lesion-related changes. The ROI-aware pipeline using RGB blue and LAB L* and a* channels reduced background-related activation and improved the spatial confinement of model attention within the fruit ROI, although performance gains and deletion-based sensitivity were architecture dependent. These findings indicate that symptom-based RGB classification combined with ROI-aware preprocessing and explainable model analysis can provide a useful controlled-condition framework for monitoring visible gray mold infection progression in postharvest kiwifruit. Future studies should validate this approach using manual lesion annotations, larger datasets collected across harvest seasons, orchards, imaging conditions, and naturally infected fruit, with particular emphasis on asymptomatic and early-stage infection detection.

## Data Availability

The original contributions presented in the study are included in the article/**Supplementary Material**. Further inquiries can be directed to the corresponding author.
