# Supplementary material for: Physiological and image-based characterization of Botrytis cinerea infection progression in postharvest ‘Haegeum’ kiwifruit using interpretable deep learning
Source: Front Plant Sci. 2026 Jul 27;17:1894572. doi: 10.3389/fpls.2026.1894572 (PMC13454441; doi:10.3389/fpls.2026.1894572)
Supplement: Supplementary file 1 [file DataSheet1.pdf]

Supplementary Material_Figures

Physiological and Image-based Characterization of *Botrytis cinerea* Infection Progression in Postharvest 'Haegeum' Kiwifruit Using Interpretable Deep Learning

Joonggon Kim[1†], Sang-Yeon Kim[2,3†], Kyeonglim Min[4], Chanyong Jeong[5], Ghiseok Kim[2,3,4], Eun Jin Lee[1,4*]

[1]Department of Agriculture, Forestry and Bioresources, College of Agriculture and Life Sciences, Seoul National University, Seoul 08826, Republic of Korea

[2]Department of Biosystems Engineering, Seoul National University, Seoul 08826, Republic of Korea

[3]Integrated Major in Global Smart Farm, Seoul National University, Seoul 08826, Republic of Korea

[4]Research Institute of Agriculture and Life Sciences, Seoul National University, Seoul 08826, Republic of Korea

[5]Department of Electrical and Computer Engineering, Seoul National University, Seoul 08826, Republic of Korea

* Correspondence:
Corresponding Author
ejinlee3@snu.ac.kr

† These authors contributed equally to this work.

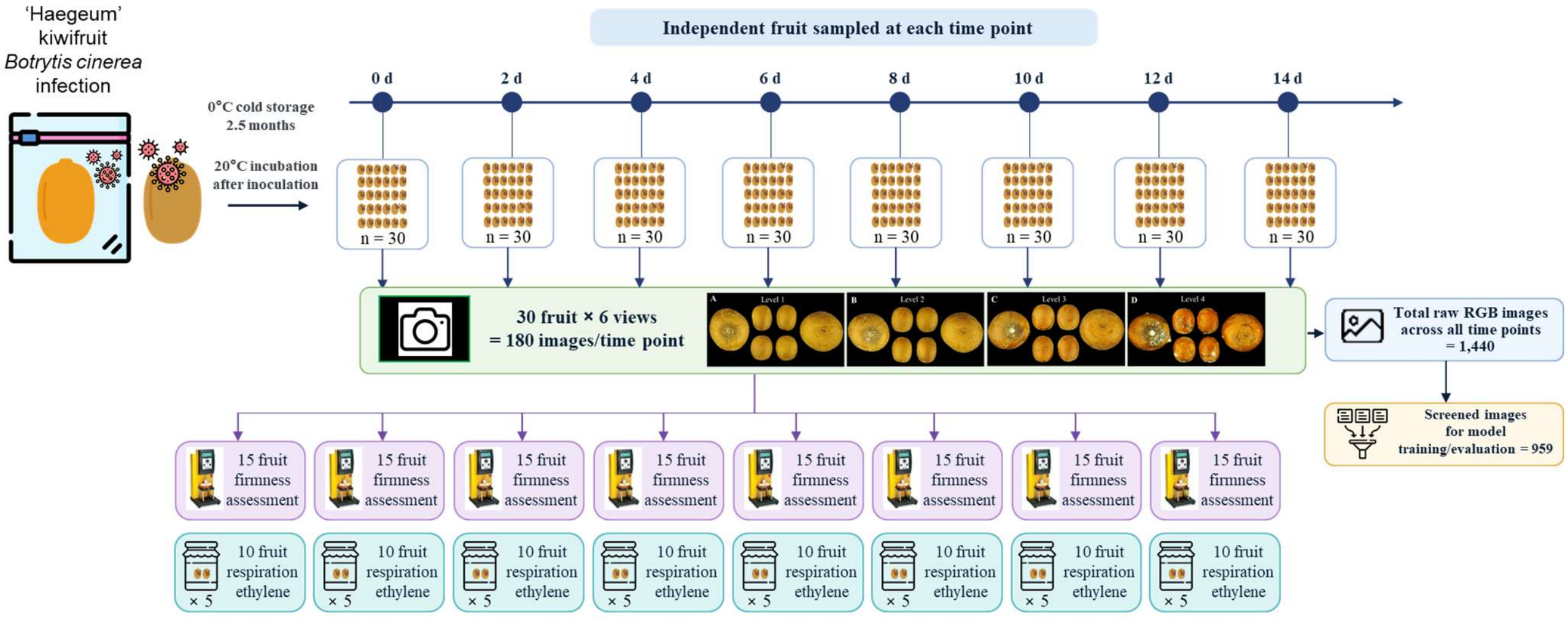

Supplementary Fig. S1. Experimental design and sample allocation for *Botrytis cinerea* inoculation, physiological measurements, and image acquisition in 'Haegeum' kiwifruit. A total of 240 fruit were allocated to eight sampling time points (0, 2, 4, 6, 8, 10, 12, and 14 d after inoculation), with 30 fruit evaluated at each time point. At each sampling time point, all fruit were photographed from six views and visually assigned to infection levels based on external symptoms. Subsequently, 15 fruit were selected for firmness measurements, 10 fruit were used for respiration rate and ethylene production analyses, and the remaining fruit were excluded from physiological measurements because their infection levels were not clearly distinguishable based on external symptoms. This imaging scheme generated 1,440 raw RGB images in total. After image-quality screening, 959 images were retained for deep-learning model training and evaluation.

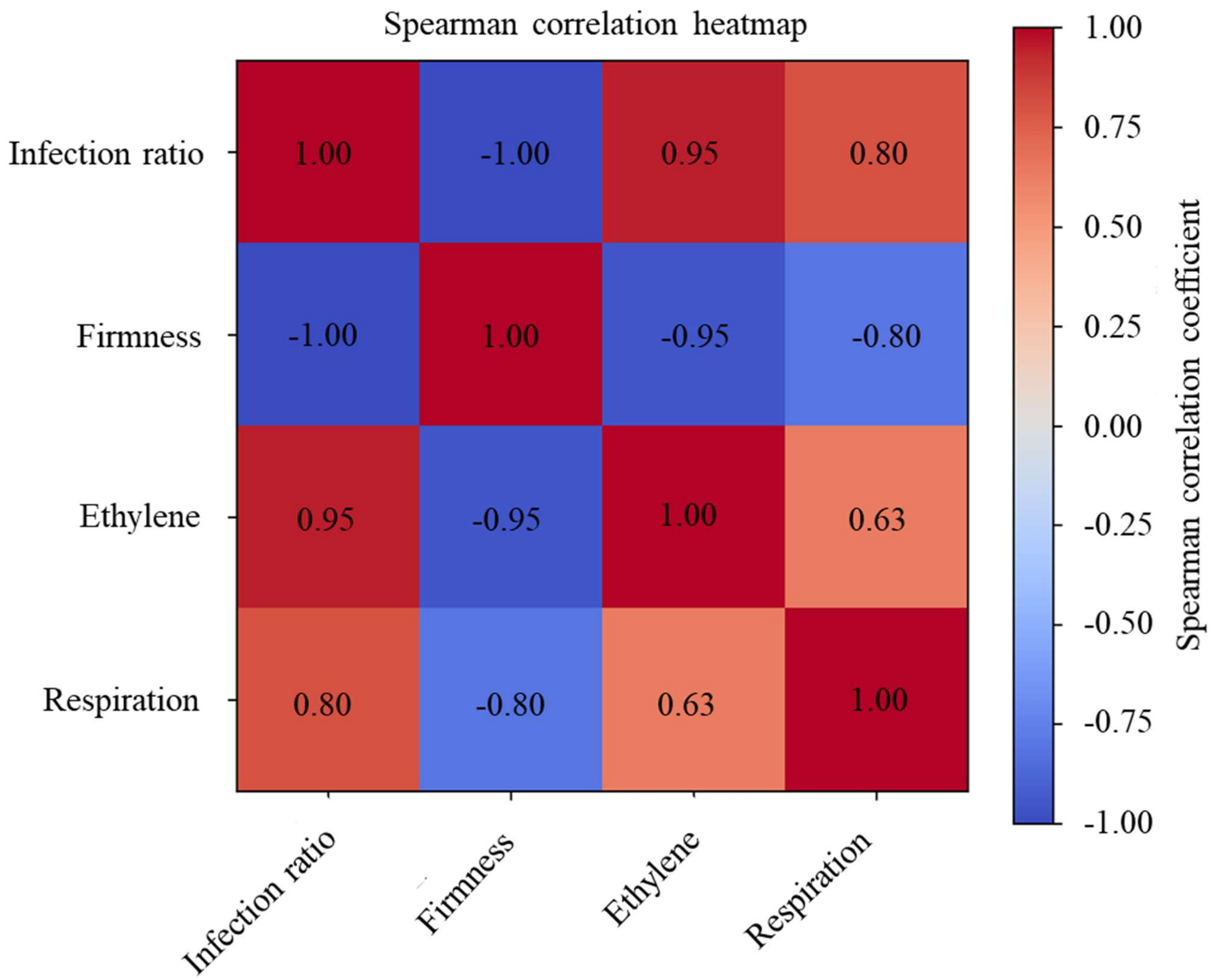

Supplementary Fig. S2. Descriptive heatmap showing associations among infection ratio, firmness, ethylene production, and respiration rate across visually assigned *Botrytis cinerea* infection levels in 'Haegeum' kiwifruit. The coefficients were calculated from level-wise mean values and are provided only as a supplementary visualization of the trends summarized in Table 1. This figure should not be interpreted as confirmatory correlation analysis or validation of infection classes.

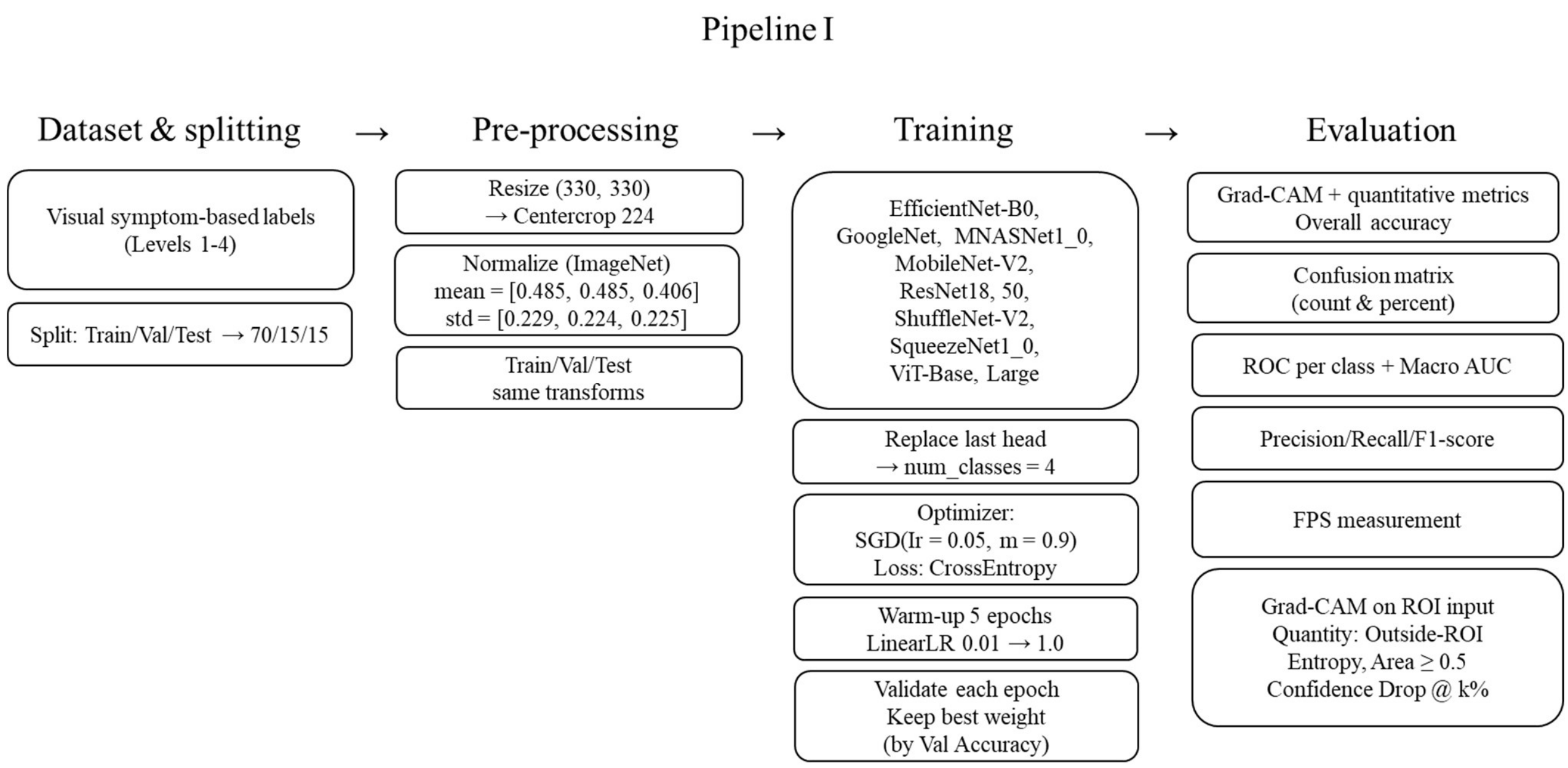

Supplementary Fig. S3. Detailed workflow of Pipeline I, the baseline RGB classification pipeline. The figure summarizes dataset splitting, RGB preprocessing, CNN training, and evaluation procedures.

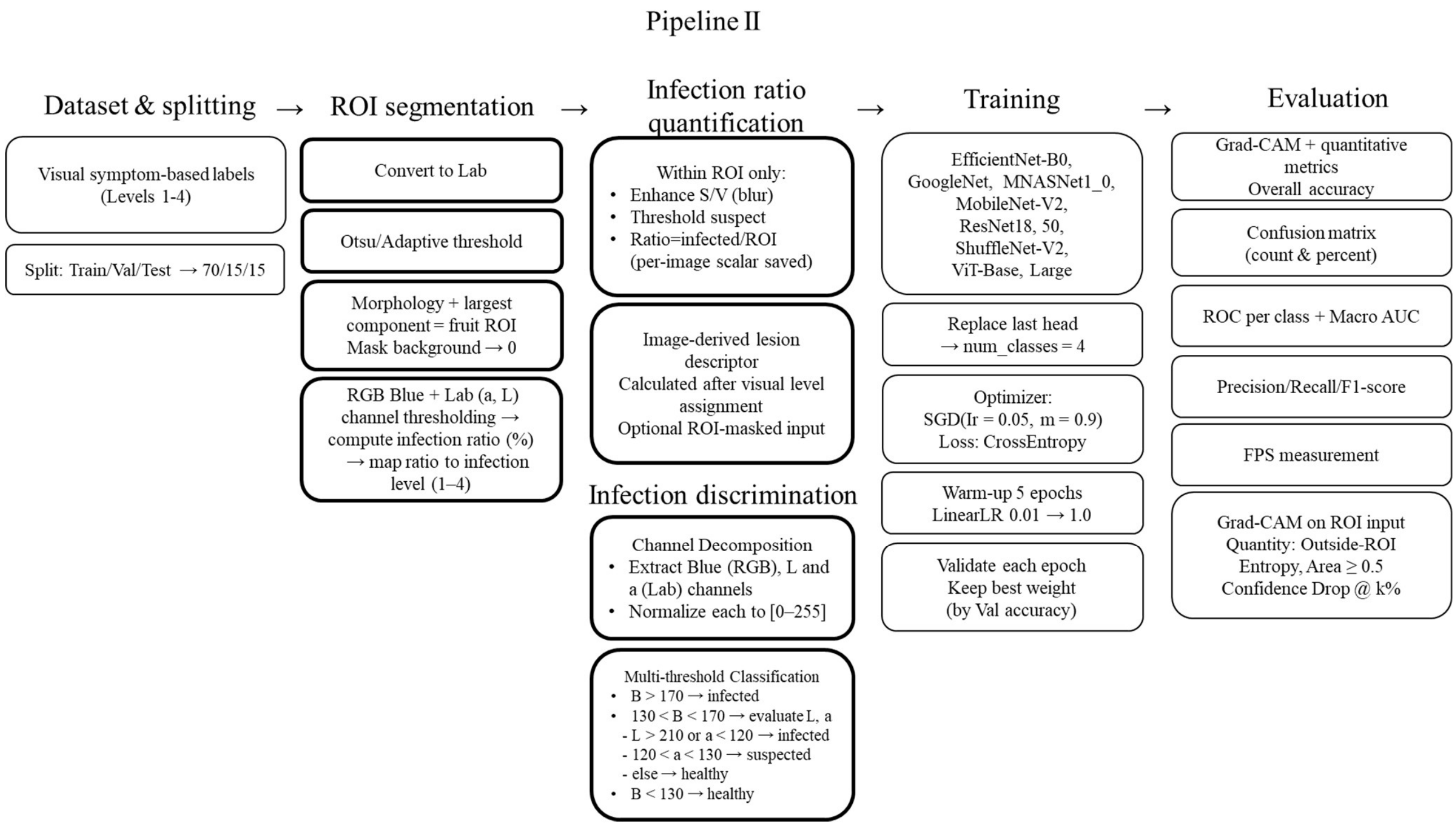

Supplementary Fig. S4. Detailed workflow of Pipeline II, the ROI-aware classification pipeline with RGB/LAB-based infection-ratio quantification. The figure summarizes fruit ROI segmentation, infection-ratio extraction, CNN training, and evaluation procedures.

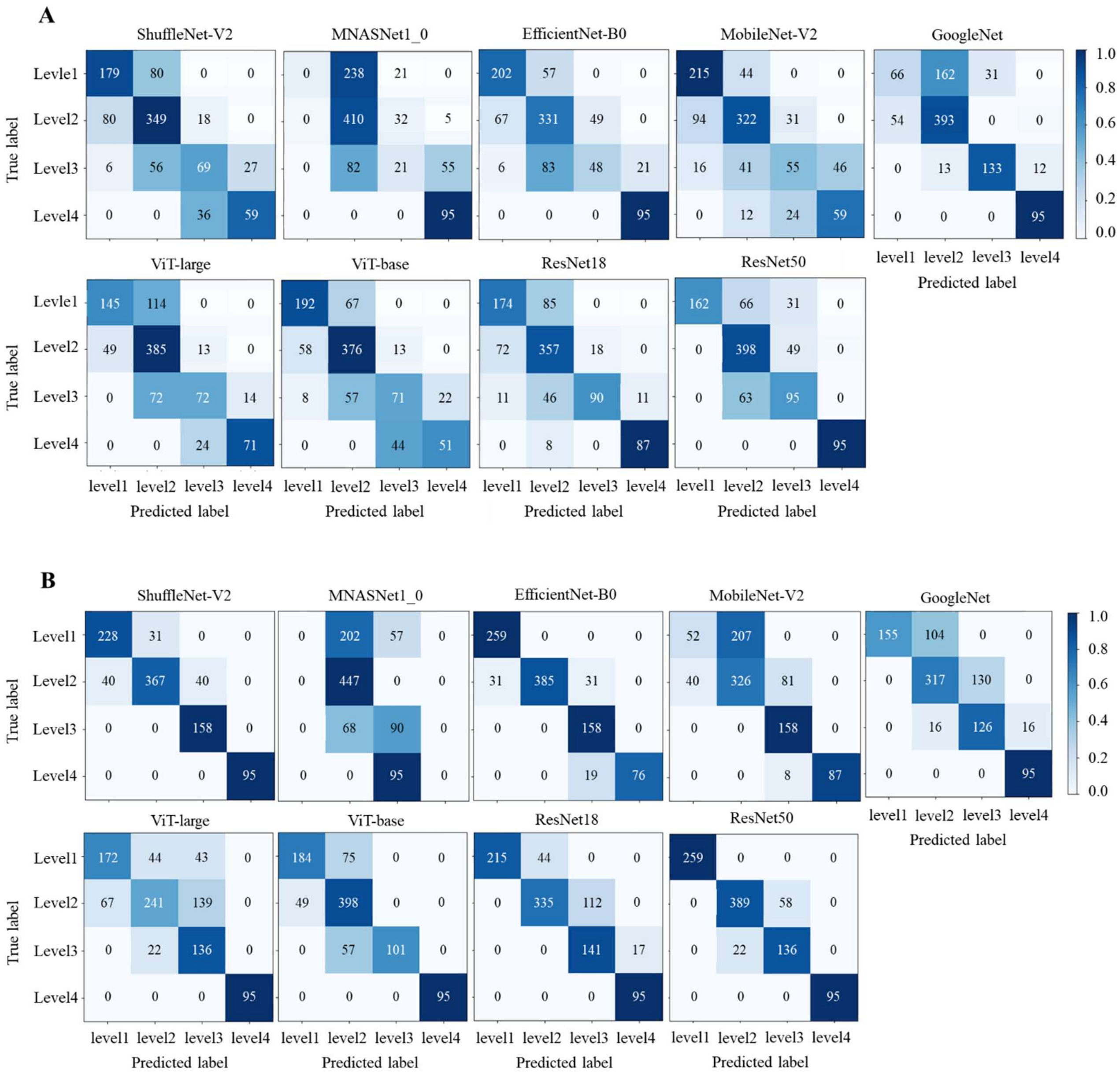

Supplementary Fig. S5. Confusion matrices for *Botrytis cinerea* infection-level classification in 'Haegeum' kiwifruit across nine deep learning backbones. (A) Pipeline I, baseline RGB pipeline. (B) Pipeline II, ROI-aware pipeline with RGB/LAB-based infection-ratio quantification. Cell values indicate the number of images assigned to each predicted class, whereas color intensity represents row-normalized prediction proportions. Rows indicate true infection levels, and columns indicate predicted infection levels.
